# The Wooten Bill

**Published:** 1895-02

**Authors:** 


THE WOOTEN BILL to regulate the practice of medicine and to prescribe the qualifications of physicians and surgeons, now before the Texas legislature, has been reported back by the committee on public health with the recommendation that it do pass, as amended by the committee. It repeals all acts in conflict with it, of course.

The amendments suggested consist of substituting the State Superintendent of Public Education, President of Board Regents State University, and the Presidents of the (three) different State Medical Associations—"regular," homeopathic [irregular], and eclectic [defective.—Daniel,]—for the Governor, Attorney-General, State Health Officer, President Board of Regents and the Dean of the School of Medicine of the Texas University, who were proposed in the bill as originally drawn, as a "Board of Medical Control;" they make also the National Association of Medicine of each of the "schools" the judge of the standing of the diplomas issued by the colleges of the respective "schools of medicine," instead of "the school of medicine to which the different colleges belong," as provided by the bill. [Of course, the school of medicine to which the colleges belong, must have a head; the committee propose the National Association of each school as the head.] This "Board of Medical Control" shall meet at Austin at least once a year, and as often thereafter as necessary; members to receive $5 per day when on attendance at meetings; the Secretary of State shall be Secretary of the Board, and shall attest all acts of the Board under seal of the State. The

Board shall prepare a list of all the reputable medical schools and colleges in the United States and foreign countries, a copy to be furnished to the clerk of each organized county in Texas, on or before the first of October each year, and as often as the Board may issue a revised list. Lists shall be open to public inspection.

By reputable colleges is meant one whose regular course of medical instruction embraces at least three full sessions of at least three months each, exclusive of special summer courses, and which is otherwise accredited as reputable by the National Association of Medicine of the school to which it belongs.

Before any person shall be permitted to practice medicine or surgery in any of their branches, he shall exhibit his diploma; the clerk shall examine it and satisfy himself that it is genuine and that the person presenting it is the identical person to whom it was originally issued; thereupon he shall issue to the applicant a certificate that the diploma has been so exhibited to him and approved. [Just how the clerk is going to determine these points is not stated in the bill.] The clerk shall keep record of all such certificates issued, with dates, and all data, and shall have a fee of $1.00 for each certificate.

This certificate then is the warrant and license to practice in any county in Texas,—the certificate to be recorded in the office of the clerk of that county to which the owner may remove; should he change location, a fee of $1.00 for recording is to be paid to the clerk of the county to which he removes, every time the doctor moves [and some of them move about as often as Willis King's "branch water men."]

All persons now holding temporary certificates or other authority to practice medicine under the present law, short of full regular certificate of satisfactory examination by the existing examining board, shall be required to surrender the same, and are not to be permitted to practice except in compliance with this act.

The act shall not apply to those who may have already qualified for practice under the act of Aug. 1, 1876, unless they hold only temporary certificates, or some "kind of authority to practice medicine short of a full certificate issued by examining boards under said act;" nor to those who "may have been regularly engaged in the practice of medicine in this State in any of its branches for five consecutive years prior to the 1st January, 1875;" nor to "females who practice midwifery strictly as such;" nor to "non-resident physicians who are graduates from respectable schools of medicine having not less than a two year's course of instruction and who have been in the actual practice for five years; *provided*, such physicians shall first apply for and obtain certificates as above required.''

The only criticism the JOURNAL has to make, besides the clerk's having to sit in judgment as to the *genuineness* of a diploma and the identity of the person presenting it, with the one to whom it was issued, is, the bill fails to define what is meant by the ''practice of medicine,'' a defect which has heretofore rendered all Texas medical laws inoperative. This is most important; it should be clearly defined. The writer is apprised of the fact that at this moment an old gentleman who does not pretend to be a doctor at all, is under arrest for selling electric belts, and is being prosecuted for violation of the medical act,--and we know of no one authorized to say to the court that selling electric belts is or is not ''practicing medicine.'' We are afraid, however, that the title of the bill will defeat it. As the JOURNAL has often pointed out, the legislature do not concede to the doctors the right to *regulate the practice.*

